# Relationship between physical activity and college students’ life satisfaction: the chain mediating effect of psychological resilience and negative emotions

**DOI:** 10.3389/fpsyg.2024.1502222

**Published:** 2025-01-14

**Authors:** Chenxin Huang, Jinfu Wang, Zixuan Chang, Jianjuan Tang

**Affiliations:** ^1^School of Physical Education, South China University of Technology, Guangzhou, Guangdong, China; ^2^School of Physical Education and Sports, Central China Normal University, Wuhan, Hubei, China

**Keywords:** college students, physical activity, psychological resilience, negative emotions, life satisfaction

## Abstract

**Objective:**

As the academic pressure, employment competition and mental health problems faced by college students are becoming more and more prominent, paying attention to and improving the quality of life and well-being of college students has become an important issue of widespread concern in all walks of life. This study focuses on the correlation between physical activity and college students’ life satisfaction.

**Methods:**

A cross-sectional survey method was applied to 326 college students, using the Physical Activity Rating Scale, the Psychological Resilience Scale, the Depression-Anxiety-Stress Scale, and the Life Satisfaction Scale. For data analysis, demographic analysis of variance, correlation analysis, and chain mediating effect test were conducted sequentially.

**Results:**

There were significant differences in psychological resilience, negative emotions, and life satisfaction by gender, and psychological resilience by grade level; there were significant correlations between physical activity and psychological resilience, negative emotions, and life satisfaction among college students (*r* = 0.541, *p* < 0.001; *r* = −0.379, *p* < 0.001; *r* = 0.435, *p* < 0.001); and psychological resilience, negative emotions had significant mediating and chain mediating effects between physical activity and life satisfaction, where the mediating effect of psychological resilience was significantly stronger than the mediating effect of negative emotions and the chain mediating effect of both.

**Conclusion:**

There was a correlation between physical activity and life satisfaction among college students, and this relationship was partially mediated by psychological resilience and negative emotions.

## 1 Introduction

Accompanied by the progress of productivity and the abundance of material resources, the question of the meaning of life and the question of self-actualization occupy an increasingly important role in the course of an individual’s life, and the results of a survey conducted by the World Health Organization (WHO) in 2023 showed that suicide has become the fourth leading cause of death for the 15–29 year old age group globally (World Health Organization, 2023). Constrained by the pressure of various external environments such as academics, work, family, etc., more and more college students gradually lose the sense of meaning in life, and “meaningless life” has become an important factor in the continuous increase of college student suicide rate in recent years (Wang, 2009). At the same time, anxiety, depression and other symptoms have become common emotional problems among college students, which may be related to the lack of daily physical activity (Liu et al., 2023; Liu, 2024). According to a survey, more than 50% of Chinese college students lack goals and motivation in their daily life (Zhang et al., 2024), which leads to a decrease in their enthusiasm for life, thus inducing suicidal, violent, and abusive behaviors. Nowadays, the consumption of the meaning of life in the complex society is calling for physical education and related research to pay attention to the contextual and experiential nature of sports, i.e., to return to the excavation of the value of physical activities (Yang and Wang, 2023). How to effectively guide college students to develop good physical activity habits and improve their life satisfaction has become an urgent issue for the government, society and academia to face and think about. This not only concerns the physical and mental health of college students, but also directly affects their future quality of life and the overall well-being of society.

Literature retrospectively found that studies on mediating variables between physical activity and life satisfaction are more extensive, and found the mediating efficacy played by factors such as self-control, self-esteem, and social support (Zhou et al., 2023; Liu et al., 2017; Dai and Yao, 2012), which verified that the pathway of physical activity’s influence on life satisfaction is a complex network involving multiple mediating variables. However, previous studies are more inclined to separate factors such as personality traits and individual emotions, failing to fully consider the possible positive or negative effects of personality traits on individual emotions. This compartmentalized treatment ignores the complex and subtle interactions between personality and mood, thus limiting our understanding of the deeper relationship between physical activity and life satisfaction. In view of this, the present study takes physical activity as the logical starting point of the research to explore the mechanism of its association with college students’ life satisfaction. In the process, we will also examine the subtle relationship between psychological resilience and negative emotions, and explore the roles of both in the relationship between physical activity on college students’ life satisfaction, with the hope of providing theoretical references and practical bases for college students to participate in physical activity in a sustainable and positive way.

### 1.1 Physical activity and college students’ life satisfaction

Physical activity, as a core concept in the field of sports, is a form of physical activity with the main purpose of promoting health, strengthening physical fitness, and regulating the body and mind (Xi, 2004). Life satisfaction, as a comprehensive psychological indicator to measure the quality of life, is the overall cognitive assessment of an individual’s life situation based on his or her own judgmental criteria for most of the time or for a certain period of time (Ci and Ning, 2013). Satisfying people’s needs for a better life has always been the eternal topic of mankind, and life satisfaction has become one of the research hotspots in the field of sports science and psychology. Many scholars have devoted themselves to exploring the mechanism of the influence of physical activity on life satisfaction, and have verified the significant effectiveness of physical activity in enhancing the life satisfaction of different groups of people.

According to the framework of embodied cognition theory, a strong link can be generated between physical experience and mental states (Liu and Wang, 2021). Since the generation of cognition is mediated by neurotransmission, the effects of physical activity on individual muscle tissue, cardiorespiratory fitness, and other bodily functions will also play a role in the cognitive level of the individual, and the enhancement of cognition, in turn, will be a key factor in improving individual life satisfaction. An experimental study revealed the direct effect of activity duration and intensity on life satisfaction, especially 40 to 60 min of moderate-intensity activity significantly increased the level of self-confidence and life satisfaction among obese elementary school students (Li et al., 2023). Another cross-sectional study further expanded the horizon by finding that extracurricular physical activity can indirectly contribute to student life satisfaction in upper elementary school through self-confidence and mental toughness (Li et al., 2022). It is worth noting that the pattern of physical activity’s impact on life satisfaction may change with age. Focusing on the elderly as a group, some studies have shown that the relationship between physical activity and life satisfaction of the elderly is an inverted U-shaped curve of “first increasing, then decreasing” (Ma et al., 2024), which may be related to the deterioration of the elderly’s physical function. As the university stage is the best period for the development of athletic ability, combined with the above analysis, we believe that there is a close relationship between physical activity and college students’ life satisfaction. Accordingly, research hypothesis H1: physical activity is positively correlated with college students’ life satisfaction.

### 1.2 The mediating role of psychological resilience

Psychological resilience is an individual’s psychological and behavioral response to changes in the external environment, reflecting an individual’s ability to face challenges and overcome adversity, and with the growth of the age of the dynamic transformation of the process includes cognitive restructuring, active adaptation, active coping, etc. (Ungar and Theron, 2020). The study concluded that there is a correlation between the level of physical activity and psychological resilience (Gao and Chen, 2022), and psychological resilience, as a concept of positive psychology, can play an important role in improving life satisfaction (Ouyang et al., 2017).

On the one hand, physical activity can have a positive impact on brain structure and function (Gao and Fan, 2020), realizing top-down self-regulation and thus enhancing the level of psychological resilience in adolescents. An empirical study has shown that physical activity helps to strengthen students’ psychological resilience and develop their problem-solving ability and focus on tasks (Wang and Bu, 2023). On the other hand, positive psychological traits such as optimism and resilience, as important indicators of psychological resilience, help individuals remain robust and adaptive in the face of life uncertainty. A longitudinal tracking study has shown that psychological resilience is a protective factor for quality of life, and that the process of physical activity can reduce the perception of psychological symptoms in secondary school students through energy consumption, emotional release, and will sharpening, which in turn enhances their positive perception of quality of life (Dong et al., 2023). Combined with the above analysis, we believe that physical activity plays a crucial role in enhancing individual psychological resilience, and the enhancement of psychological resilience becomes a key bridge to improve life satisfaction. Accordingly, the research hypothesis H2 is proposed: psychological resilience has a mediating effect between physical activity and college students’ life satisfaction.

### 1.3 The mediating role of negative emotions

Negative emotions are individuals’ subjective experience of unpleasant emotions such as depression and anxiety (Zhang et al., 2004). At the behavioral or pathological level, the accumulation of negative emotions can negatively affect an individual’s physical and mental health and social adaptability (Liu and Zhang, 2024). Among the many studies on negative emotions, scholars have paid close attention to the relationship between physical activity and negative emotions, and have intervened in individuals’ negative emotions through regular extracurricular physical activity with a certain level of intensity, verifying the positive impact of physical activity on individuals’ experience of negative emotions (Gong et al., 2019).

A cross-sectional study found that insufficient physical activity leads to depressive symptoms in college, i.e., a decrease in physical activity is accompanied by an increased risk of depressive symptoms (Liu, 2020). In addition, an experimental study has shown that physical activity of different exercise intensities and different organizational forms can effectively reduce test anxiety levels in adolescents (Zhang and Jiang, 2010). From the biological mechanism, animal and human studies have demonstrated that physical activity significantly increases the expression level of PGC-1α in skeletal muscle.PGC-1α, as a transcriptional coactivator, is able to effectively regulate peripheral kynurenine metabolism, inflammatory response and endocrine function in skeletal muscle. It is also involved in regulating the concentration of neurotrophic factors, the level of glucocorticoid hormones, affecting the morphology and structure of specific parts of the central nervous system, and regulating the release of pro-inflammatory cytokines, inducing neurogenesis in the hippocampal region of the central nervous system, effectively activating the central nervous system, thus alleviating negative emotions and prompting the individual to maintain a positive attitude towards life (Wang et al., 2023). Combined with the above analysis, we believe that physical activity has a significant role in reducing individuals’ negative emotional experience, and this reduction of negative emotions is an important way to enhance life satisfaction. Accordingly, research hypothesis H3 is proposed: negative emotions have a mediating effect between physical activity and college students’ life satisfaction.

### 1.4 Construction of the chain mediator model

Some studies have confirmed that psychological resilience has been shown to inversely correlate with negative emotions (He et al., 2022). Specifically, in the face of stressful events, individuals with higher levels of psychological resilience are more likely to maintain a positive and optimistic attitude, and are able to face their own shortcomings and deficiencies (Zhang et al., 2016). In this context, individuals are able to recover from failure in a short period of time and avoid the buildup of anxiety, depression, and other emotions, and curb the narrow cognition triggered by them in a timely manner, so as to respond positively and effectively to the problem. On the other hand, they tend to magnify the negative information in their lives, making them sensitive to interpersonal interactions. Therefore, groups with higher levels of psychological resilience are more likely to avoid negative emotions.

Several empirical studies have shown that psychological resilience can act as a protective factor when individuals face environmental setbacks and play a moderating role between stressors and negative emotions (Sarkar and Fletcher, 2014). It is worth noting that the personality trait of psychological resilience is not genetically predetermined; it is a potential that exists in every adolescent and can be enhanced through training (Xiao et al., 2022). Currently, most of the depression prevention programs implemented in domestic and international studies are based on cognitive-behavioral interventions, in which encouraging adolescents to participate in physical activities is one of the interventions (Fu and Tang, 2024). In addition, for the special group of highland military personnel, some surveys have shown that officers and soldiers with health literacy have higher average scores of psychological resilience and positive coping. On the contrary, officers and soldiers with poor health literacy have higher mean scores of negative emotions and negative coping (Chen et al., 2018). In summary, although existing studies have revealed the associations between physical activity and psychological resilience, psychological resilience and negative affect, and negative affect and life satisfaction, respectively, in-depth explorations on the intrinsic mechanisms and interactions among these four variables are still insufficient. In this study, we propose to use physical activity as the independent variable, life satisfaction as the dependent variable, and psychological resilience and negative emotions as the chain mediation variables. Accordingly, the research hypothesis H4 is proposed: psychological resilience and negative emotions have a chain mediating effect between physical activity and college students’ life satisfaction.

**Figure 1:**
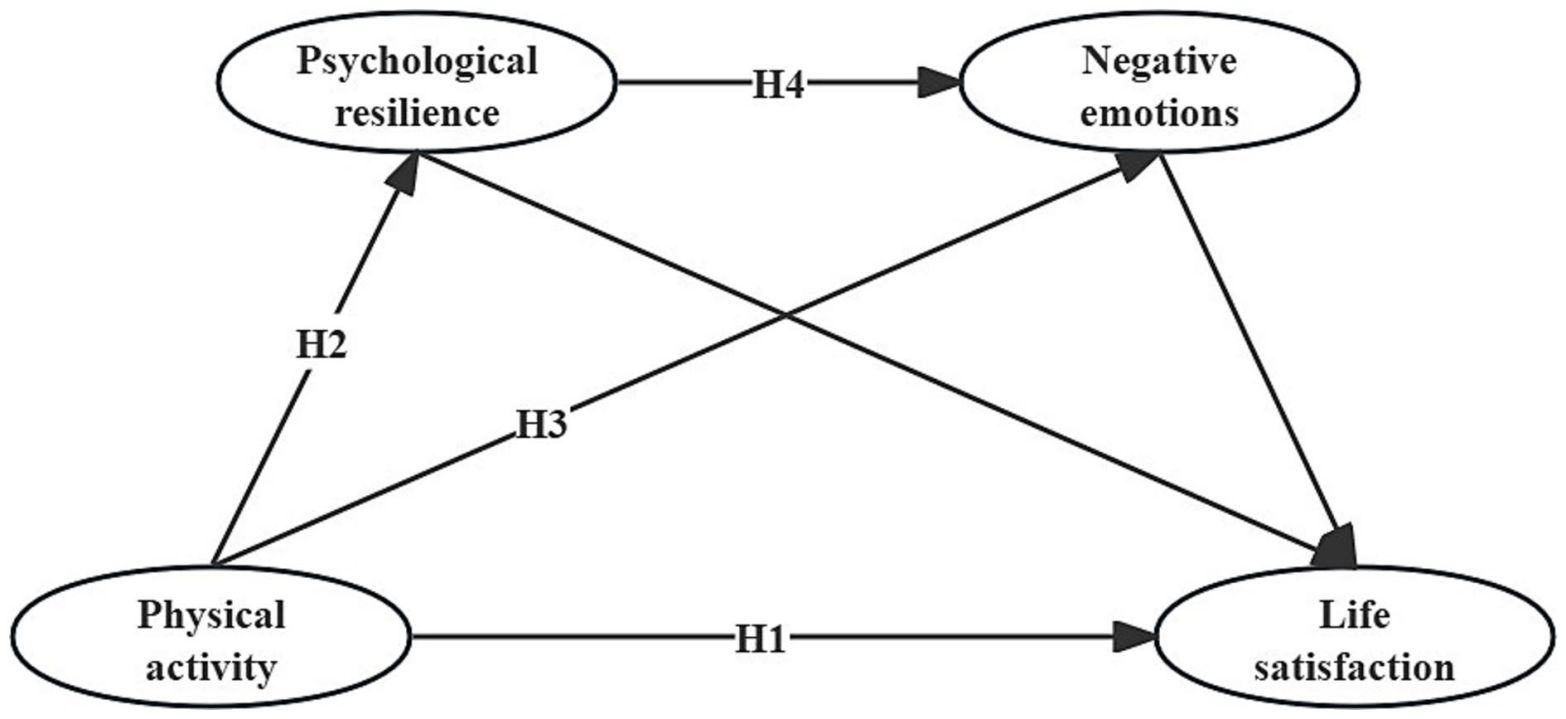
Hypothetical model diagram of physical activity affecting college students’ life satisfaction.

Based on the exploration of the relationship between different variables, we introduced for the first time psychological resilience and negative emotion factors between physical activity and life satisfaction, and tried to construct a chain mediation hypothesis model between physical activity and college students’ life satisfaction (e.g., Figure 1).

## 2 Materials and methods

Permission must be obtained for use of copyrighted material from other sources (including the web). Please note that it is compulsory to follow figure instructions.

### 2.1 Participants

GPower 3.1 was used to estimate the *a priori* sample size for this study. The specific settings are as follows: *f* = 0.25; *α* = 0.05; power (1-*β*) = 0.95, resulting in a minimum sample size of 210. This study adopted a convenience sampling method, selecting students from three colleges and universities in Guangdong Province as the target respondents, and inviting 120 students from each college and university to fill in the online questionnaire to ensure that the sample size of the study had sufficient statistical validity. In screening participants, we established clear inclusion and exclusion criteria to ensure the accuracy and generalizability of the study. Inclusion criteria were: full-time college students (undergraduate and graduate), no physical disabilities, able to engage in physical activity, and had not previously participated in a similar study. Exclusion criteria included failure to meet inclusion criteria, response anomalies (such as filling out answers consistently or taking less than 1 min), and failure to complete the questionnaire. Questionnaire Star was chosen as the platform for collecting the questionnaires, which is similar to Amazon Mechanical Turk. Before starting the questionnaire, the subject will be informed of the anonymity, confidentiality and purpose of the survey. After screening according to the above inclusion and exclusion criteria, the final number of valid questionnaires was determined to be 326, with a valid return rate of 93%. In the gender distribution of the samples, there were 166 males, accounting for 50.9%, and 160 females, accounting for 49.1%. In the distribution of the sample grade, there were 83 people in the first year, accounting for 25.5%; 80 people in the second year, accounting for 24.5%; 71 people in the third year, accounting for 21.8%; 49 people in the fourth year, accounting for 15%; and 43 people in the master’s degree and above, accounting for 13.2%.

### 2.2 Measures and procedures for data collection

#### 2.2.1 Physical activity

The Physical Activity Rating Scale revised by Liang (1994) was used to measure college students’ participation in physical activity in the past month. The scale was assessed in three main aspects: intensity, duration, and frequency of activity, and a 5-point scale was used. The scores for activity intensity and frequency range from 1 to 5, and the scores for duration range from 0 to 4. The score is calculated as “activity intensity × duration × activity frequency,” and the total score of the scale ranges from 0 to 100. Higher scores indicate higher levels of physical activity among college students. The Cronbach’s alpha reliability coefficient of the scale questionnaire in this study was 0.771.

#### 2.2.2 Psychological resilience

The Resilience Scale revised by Yu and Zhang (2007), was used to measure the degree of psychological resilience of college students. The scale consists of three dimensions: strength, resilience, and optimism, with a total of 25 entries, and a 5-point scale, with scores ranging from 0 to 4, from “never” to “always,” and the total score ranging from 0 to 100. The higher the score, the higher the level of psychological resilience of college students. The Cronbach’s alpha reliability coefficient of the scale questionnaire in this study was 0.932, and the Cronbach’s alpha reliability coefficients of the three dimensions were 0.930, 0.868, 0.952, respectively. At the same time, the questionnaire was subjected to a validation factor analysis, and the results showed that the model’s various fit indices were good, x^2^/df = 1.306, RMSEA = 0.031, NFI = 0.938, RFI = 0.931, CFI = 0.985, PCFI = 0.893, which indicates that the form has good construct validity.

#### 2.2.3 Negative emotions

The short-form version of the Depression Anxiety Stress Scales revised by Gong et al. (2010), was used to measure the status of negative emotions in college students. The scale contains three dimensions of depression, anxiety, and stress, with a total of 21 entries, and adopts a 4-level scoring system, with scores ranging from 0 to 3, from “does not meet” to “fully meet,” and the total score of the scale ranges from 0 to 63 points. The higher the score, the stronger the negative emotional experience of college students. The Cronbach’s alpha reliability coefficient of the scale questionnaire in this study was 0.920, and the Cronbach’s alpha reliability coefficients of the three dimensions were 0.935, 0.935, 0.938, respectively. Meanwhile, the questionnaire was subjected to a validation factor analysis, and the results showed that the model’s various fit indices were good, x^2^/df = 1.033, RMSEA = 0.010, NFI = 0.964, RFI = 0.959, CFI = 0.999, PCFI = 0.885, which indicates that the form has good construct validity.

#### 2.2.4 Life satisfaction

The Satisfaction With Life Scale developed by Diener et al. (1985), was used to measure college students’ perceptions of their level of satisfaction with life. The scale is a unidimensional scale with a total of 5 entries and a 7-point scale, with scores ranging from 1 to 7 from “Strongly Disagree” to “Strongly Agree,” and the total score of the scale ranges from 5 to 35 points. The higher the score, the higher the college students’ satisfaction with life. The Cronbach’s alpha reliability coefficient of the scale questionnaire in this study was 0.953.

### 2.3 Data analysis

The study used SPSS 26.0 to statistically analyze the data. First, a common method bias test was conducted, followed by analysis of variance of physical activity, psychological resilience, negative emotions, and life satisfaction among college students with different demographic characteristics, followed by correlation analysis of physical activity, psychological resilience, negative emotions, and life satisfaction, and finally, the proposed chain mediation model was tested using the PROCESS macro program developed by Hayes.

## 3 Results

### 3.1 Common method bias test

Common methodological bias refers to the fact that due to common systematic errors in data collection methods, measurement instruments, or research design, the measurement results do not fully reflect the true values of the variables measured, which in turn leads to spurious correlations between the study variables. To avoid common method bias, coded anonymous assessments were used during administration to procedurally control for sources of common method bias. All test items were examined using Harman’s one-way analysis of variance. The results showed that there were eight factors with eigenroots greater than 1. The variance explained by the first factor was 30.34, which was less than the critical threshold of 40% (Xiong et al., 2012), indicating that there was no common method bias in this study.

### 3.2 Analysis of differences in demographic variables

The scores of physical activity, psychological resilience, negative emotions and life satisfaction of college students with different demographic characteristics were compared between two groups by t-test and between multiple groups by analysis of variance (ANOVA). The results of the analysis showed that there were statistically significant gender differences in psychological resilience, negative emotions and life satisfaction of college students, and that male college students were better than female college students in psychological resilience and life satisfaction, and female college students were more likely to be in negative emotions than male college students. In addition, the psychological resilience of college students is statistically significant in the difference of grade, and the overall trend is that the higher the grade, the higher the level of psychological resilience of college students (see Table 1).

**Table 1:** Differences in gender and grade.

	Groups	Statistical value	Physical activity	Psychological resilience	Negative emotions	Life satisfaction
Gender	Male (166)		34.49 ± 24.40	64.18 ± 18.30	35.67 ± 12.48	21.34 ± 6.69
	Female (160)		31.34 ± 24.02	59.06 ± 18.45	38.70 ± 12.31	19.71 ± 6.22
		*t*	1.174	2.514	−2.203	2.278
		*p*	0.241	0.012	0.028	0.023
Grade	Freshman year (83)		29.48 ± 24.11	57.72 ± 19.26	36.86 ± 13.36	19.29 ± 6.78
	Sophomore (80)		33.40 ± 24.16	64.15 ± 17.19	37.55 ± 11.42	20.88 ± 6.04
	Junior (71)		33.10 ± 25.75	59.72 ± 19.49	37.51 ± 13.24	21.17 ± 6.54
	Senior (49)		30.49 ± 19.42	61.69 ± 17.21	38.47 ± 11.61	19.84 ± 6.57
	Master’s degree (43)		41.35 ± 25.90	67.86 ± 17.76	35.95 ± 12.47	22.07 ± 6.45
		*F*	1.870	2.756	0.514	1.740
		*p*	0.115	0.028	0.725	0.141

### 3.3 Correlation analysis of physical activity, psychological resilience, negative emotions, and life satisfaction

Given the significant influence of gender and grade level on the key variables of psychological resilience and life satisfaction, this study further used partial correlation analysis to include these two important demographic variables as covariates. The results of the analysis showed that there was a significant positive correlation between physical activity and college students’ life satisfaction, a significant positive correlation with psychological resilience, and a significant negative correlation with negative emotions. Meanwhile, there was a significant negative correlation between psychological resilience and negative emotions, a significant positive correlation with college students’ life satisfaction, and a significant negative correlation between negative emotions and college students’ life satisfaction (see Table 2).

**Table 2:** Descriptive statistics and correlation coefficients of the variables.

Variable	M ± SD	Physical activity	Psychological resilience	Negative emotions	Life satisfaction
Physical activity	32.95 ± 24.23	1			
Psychological resilience	61.67 ± 18.52	0.541***	1		
Negative emotions	37.16 ± 12.47	−0.379***	−0.522***	1	
Life satisfaction	20.54 ± 6.50	0.435***	0.568***	−0.559***	1

*p < 0.05, **p < 0.01, ***p < 0.001, same below.

Due to the significant correlation between the variables, there may be a problem of multicollinearity, which can lead to unstable outcome results. Therefore, the predictor variables in each equation were standardized and tested for covariance. The results showed that all variance inflation factors VIF (1.00–1.73) were less than 5 and there was no multicollinearity problem in the data.

### 3.4 Chain mediating effect test

Since there were significant correlations between physical activity, psychological resilience, negative emotions, and life satisfaction, and there was no problem of multiple covariance, the statistical requirements for testing the direct and indirect effects of physical activity and college students’ life satisfaction were met. The data were normalized and the PROCESS macro in SPSS was used to control for the demographic variables (gender and grade) to test the chain mediating effects of psychological resilience and negative emotions between physical activity and college students’ life satisfaction at 95% confidence intervals. PROCESS macro is a plug-in for analyzing mediating and moderating effects. The settings were as follows: model 6 was chosen; X = physical activity, M1 = psychological resilience, M2 = negative emotions, Y = life satisfaction; Bootstrap Sample = 5,000.

**Figure 2:**
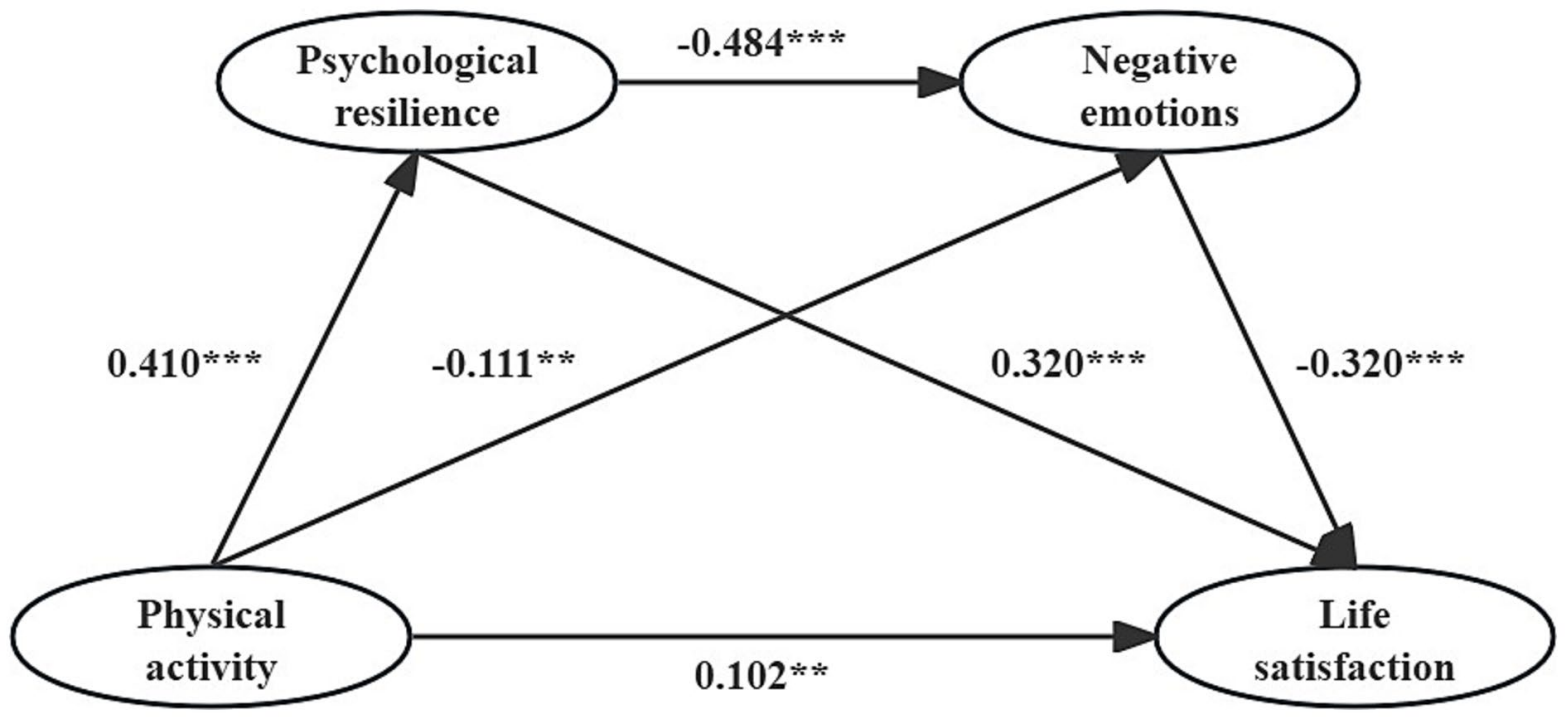
Standardized path of physical activity and life satisfaction of college students.

The results of regression analysis showed (Table 3) that there was a significant positive relationship between physical activity and college students’ life satisfaction. After psychological resilience and negative emotions were jointly included in the regression equation, physical activity still had a significant positive relationship with college students’ life satisfaction, but the effect size was weakened, which indicated that psychological resilience and negative emotions played a partial mediating role between physical activity and life satisfaction. Specifically, physical activity showed a significant positive relationship with college students’ psychological resilience and a significant negative relationship with negative emotions. Meanwhile, psychological resilience showed a significant negative relationship with negative emotions, and both of them showed significant positive and negative relationships with college students’ life satisfaction, respectively. The specific path model is shown in Figure 2.

**Table 3:** Regression analysis of variable relationships.

Variable	Life satisfaction		Psychological resilience		Negative emotions		Life satisfaction	
	*β*	*t*	*β*	*t*	*β*	*t*	*β*	*t*
Gender	−0.070	−1.897	−0.074	−2.152*	0.042	1.132	−0.022	−0.687
Grade	0.031	0.889	−0.039	1.238	0.039	1.135	0.024	0.842
Physical activity	0.332	8.668***	0.410	11.548***	−0.111	−2.420**	0.102	2.636**
Psychological resilience					−0.484	−7.998***	0.320	5.773***
Negative emotions							−0.320	−6.861**
*R*	0.457		0.561		0.544		0.666	
*R* ^2^	0.208		0.315		0.296		0.443	
*F*	28.252		49.444		33.786		50.989	

The results of the mediation effect analysis showed (Table 4) that psychological resilience and negative emotions mediated the relationship between physical activity and life satisfaction of college students. Further refinement revealed that this mediating effect was achieved through three significant paths: (1) physical activity→psychological resilience→life satisfaction, the indirect effect value of its path was 0.131, and the 95% confidence interval did not contain 0, indicating that the indirect effect of the mediator variable of psychological resilience was significant and accounted for 39.46% of the total effect; (2) physical activity→negative emotions→life satisfaction, the indirect effect value is 0.036, 95% confidence interval does not contain 0, indicating that the indirect effect of the mediating variable of negative emotion is significant, accounting for 10.84% of the total effect; (3) physical activity→psychological resilience→negative emotion→life satisfaction, the indirect effect value of its path is 0.064, 95% confidence interval does not contain 0, indicating that the chain mediating effect of both psychological resilience and negative emotion is significant, accounting for 19.28% of the total effect. Comparing the indirect effects of the 3 pathways, the results showed that the indirect effect of psychological resilience was significantly higher than the indirect effect of negative emotions. The chain mediating effect between both negative affect and psychological resilience was significantly lower than the indirect effect of psychological resilience and not significantly different from the indirect effect of negative affect.

**Table 4:** Results of Bootstrap mediated effects analysis.

Impact pathways	Effect	BootSE	BootLLCI	BootULCI	Effect size ratio
Total effect	0.332	0.038	0.257	0.408	
Direct effect	0.102	0.039	0.026	0.178	
Total indirect effect	0.230	0.027	0.179	0.284	69.28%
Ind1 (DL → TX → MY)	0.131	0.027	0.080	0.187	39.46%
Ind2 (DL → QX → MY)	0.036	0.015	0.007	0.066	10.84%
Ind3 (DL → TX → QX → MY)	0.064	0.012	0.042	0.090	19.28%
C1 (Ind1-Ind2)	0.096	0.035	0.028	0.166	
C2 (Ind1-Ind3)	0.068	0.032	0.005	0.130	
C3 (Ind2-Ind3)	−0.028	0.020	−0.070	0.010	

DL denotes physical activity; TX denotes psychological resilience; QX denotes negative emotions; MY denotes life satisfaction.

## 4 Discussion

This study explored the association between physical activity and college students’ life satisfaction, and confirmed the mediating and chaining effects played by psychological resilience and negative emotions. These findings corroborate our four previously proposed hypotheses and provide a new perspective and explanatory framework for further understanding the mechanism of the association between physical activity and college students’ life satisfaction.

### 4.1 The relationship between physical activity and college students’ life satisfaction

As predicted by Hypothesis H1, the study found that there is a positive relationship between physical activity and college students’ life satisfaction, i.e., the higher the level of physical activity of college students, the higher the level of life satisfaction they have. This is consistent with previous research results (Chmelík et al., 2023). Long-term regular physical activity of college students can enhance cardiorespiratory fitness, strengthen muscle strength, and improve immune system function, thus reducing the risk of physical or mental illness (Imayama et al., 2013). Good physical and mental health development is the basic guarantee for college students to cope with academic and life challenges, and is important for improving college students’ life satisfaction. In addition, physical activity is usually a social activity, and its unique value lies in its ability to make college students temporarily get rid of the pressure of schooling and employment in the interaction and communication, and achieve effective diversion of attention, which can bring physical and mental satisfaction and pleasure, and effectively alleviate anxiety and depression (Zhu et al., 2023). At the same time, physical activity has a regulating effect on the posture of college students, and continuous physical activity will promote the improvement of college students’ body shape. A study has shown that physical activity can improve the asymmetrical muscle strength of the spine on both sides of the spine, such as the erector spine muscles, increase the proprioception, muscle control and coordination of college students, and gradually restore the spine to its normal position (Wang W. J. et al., 2022). And the correction and improvement of body shape will bring about the improvement of overall temperament, and this improvement of body image and temperament helps college students better adapt to changes in the external environment and indirectly improves life satisfaction (Gao et al., 2007).

### 4.2 Independent mediating effect of psychological resilience

As predicted by hypothesis H2, psychological resilience was found to mediate the relationship between physical activity and college students’ life satisfaction. This is consistent with previous research results (Kong et al., 2015). Currently, physical activity has become an indispensable part of college students’ campus life. For individuals, long-term participation in physical activity has the effect of enhancing self-confidence and resistance to frustration (Gong et al., 2020). This process not only promotes the physical and mental health development of college students, but also strengthens their resilience and self-confidence in the face of challenges. Especially in basketball, football, rugby and other large-scale team sports, the effect is more significant. The cooperation and communication facilitated by team sports help college students establish and maintain good interpersonal relationships (Yang and Xiang, 2021). Self-confidence, interpersonal relationships and other such factors are related to the internal and external formation factors of individual psychological resilience, which enhance the psychological resilience level of college students to a greater extent. On the other hand, according to the self-control power model, college students will consume psychological energy during physical activity, but this energy will also be continuously restored and gradually improved, enhancing self-control in a virtuous circle (Tan et al., 2012). The enhancement of self-control power changes the basic perceptual ability of college students, showing stronger psychological resilience and social adaptability, and better tolerance and acceptance of complex changes in the surrounding environment (Yu et al., 2022), which is undoubtedly a key factor in the continuous improvement of college students’ life satisfaction.

### 4.3 Independent mediating effect of negative emotions

As predicted by hypothesis H3, negative emotions were found to mediate the relationship between physical activity and college students’ life satisfaction. This is consistent with previous research results (Liu et al., 2021). Heart flow theory shows that the “flow” state caused by physical activity plays a significant role in reducing negative emotions such as stress and anxiety, and improving personal well-being (Guo, 2015). From the perspective of behavioral science, physical activity as a medium for individuals to provide a space for interaction, college students through communication with peers to produce empathy and form group identity, and timely curb the occurrence of negative emotions. Some intervention studies have shown that moderate to high-intensity aerobic activity three or more times per week has a more positive effect on alleviating negative emotions and promoting life satisfaction (Tao et al., 2023). From the perspective of activity human science, when the human body is in the activity state, the body releases a large amount of neurotransmitter dopamine, and the release of dopamine can make people refreshed, energetic, and alleviate the generation of college students’ negative emotions, such as anxiety, depression, etc. (Chen and Ji, 2003). At the same time, physical activity also promotes the production of *β*-endorphin, the release of β-endorphin can act on the pituitary gland and make people feel happy, thus stimulating the cognitive thinking and emotional thinking of college students, which is conducive to enhancing the sense of satisfaction of college students’ life (Wang H. X. et al., 2022).

### 4.4 Chain mediating effect of psychological resilience and negative emotions

As predicted by hypothesis H4, it was found that psychological resilience and negative emotions play a chain mediating role in the relationship between physical activity and college students’ satisfaction. Specifically, physical activity can not only exercise individual willpower and endurance, but also cultivate the ability of college students to maintain a positive mindset in the face of adversity, so that college students can show more resilience in the face of academic pressures, challenges in life, and complex interpersonal relationships (Liu et al., 2019). Negative emotions in college students often arise from multiple pressures, such as heavy academic tasks, complex interpersonal relationships, and sudden major changes, etc. Increased psychological resilience can reduce the negative impact of adversity or traumatic events on college students, and provide a protective effect for college students in the face of negative events, which can help college students better cope with potential threats (Zhang et al., 2022). More importantly, college students with higher levels of psychological resilience show a positive bias in cognitive processing, i.e., preferential attention to positive information and avoidance attention to negative information. This cognitive bias can to some extent buffer the adverse emotional reactions brought about by negative stimuli, thus reducing the impact of negative emotions on the psychological state of college students and contributing to the high satisfaction of college students with their life state (An et al., 2022).

## 5 Implications and limitations

The study explored the chain mediating role of psychological resilience and negative emotions between physical activity and college students’ life satisfaction, making the first attempt to link these variables together and expanding previous findings to some extent. The associations between physical activity, mental toughness, negative emotions, and college students’ life satisfaction were further elucidated by analyzing the chain mediation model. Accordingly, in practice, the government should deepen top-level strategic planning to build a policy ecosystem that is conducive to college students’ extensive participation in physical activity, and ensure that solid institutional safeguards and incentives are provided for college students’ participation in physical activity. At the same time, schools, social organizations and other actors should respond positively to the government’s call to take the initiative to organize diversified sports activities, and actively advocate and encourage more female college students to participate in sports and exercise activities. Through the planning and implementation of a series of creative and effective sports activities, we can cultivate their mental toughness in the face of challenges and adversities, enhance their ability to fight against setbacks and overcome difficulties, and thus promote their better integration into college studies and social life, and gain a sense of satisfaction from them.

While exploring this study, we were faced with several limiting considerations: Firstly, although our sample encompassed college students of different genders and grades, which provided a favorable condition for obtaining diverse perspectives. However, we must be recognized that this may lead to issues of selection bias due to the use of convenience sampling methods. This limitation somewhat reduces the generalizability of the findings and may not be representative of all college student populations. Secondly, the study relied primarily on self-reported data collection methods, which are susceptible to subjective factors and may introduce certain cognitive biases or recall biases, affecting the objectivity and accuracy of the data. Thirdly, the study adopted a cross-sectional research design, which can reveal the current situation at a certain point in time, but it is difficult to capture the dynamic relationship between variables over time, especially the inference of long-term effects and causality is limited. To address the above limitations, subsequent studies should consider using random sampling methods and larger sample sizes to improve the validity of the results. Meanwhile, the assessment tool should be optimized to make its data reflect the actual situation more accurately, and longitudinal design or experimental study should be used, which is expected to be supplemented and explored by subsequent studies and more scholars.

## 6 Conclusion

This study systematically explored the correlation between physical activity and college students’ life satisfaction, and deeply analyzed the underlying internal mechanisms. By constructing a chain mediation model, we further elucidated the relationship between physical activity and college students’ life satisfaction. However, it is undeniable that this result of the current study needs to be further verified for its validity and reliability through more types of studies, including longitudinal studies, experimental studies, and so on.

## Data Availability

The original contributions presented in the study are included in the article/supplementary material, further inquiries can be directed to the corresponding author.
